# A COMPREHENSIVE ALZHEIMER’S AND DEMENTIA CARE PROGRAM (ADCP) FOR PATIENTS AND CAREGIVERS

**DOI:** 10.1093/geroni/igac059.2778

**Published:** 2022-12-20

**Authors:** Madieska Ramirez, Prabhash Kakarla, Marzena Gieniusz, Jessica Alvarez, Chris Nouryan, Edith Burns

**Affiliations:** Hofstra-Northwell, New Hyde Park, New York, United States; Hofstra University, New Hyde Park, New York, United States; Hofstra-Northwell, New Hyde Park, New York, United States; Hofstra-Northwell, New Hyde Park, New York, United States; Zucker School of Medicine at Hofstra Northwell, Uniondale, New York, United States; Zucker School of Medicine at Hofstra Northwell, Manhasset, New York, United States

## Abstract

**Background:**

Alzheimer’s and related dementias (ADRD) are progressive and associated with behavioral and psychological symptoms (BPSD) contributing to caregiver stress. We report initial impact of implementing UCLA-designed ADCP to support ADRD patients and caregivers. The program centralizes care, utilizing a nurse dementia-care specialist.

**Methods:**

Prospective repeated measures of ADRD patient/caregiver dyads from 3/2021–7/2022. Measures: BPSD, ADL/IADL, cognition, and caregiver burden, distress and depression.

**Results:**

Total of 154 patient/caregiver dyads enrolled to-date. 42 have been enrolled for one-year: 23 with complete data, 5 pending follow-up, 14 disenrolled (2 expired, 3 to hospice, 2 moved, 7 lost contact). For 23 patients with baseline and follow up, Neuropsychiatric Inventory Questionnaire decreased from 16.3 to 13.1 (p=0.170), Patient Health Questionnaire-9 (PHQ-9) for caregivers decreased (3.5 to 2.2, p=0.044), Montreal Cognitive Assessment decreased from 11.3 to 8.4 (p=0.045), and Dementia Burden Scale (DBS) showed nonsignificant decrease, 20.6 to 18.4 (p=0.312). Cornell Scale for Depression in Dementia was unchanged, 5.67 to 5.14 (p=0.380), and ADL and IADL decreased one year after enrollment, from 4.05 to 2.36 (p < 0.01) and 1.38 to 0.238 (p < 0.01) respectively.

**Conclusion:**

Although attrition rate was high for the small sample (1/3 of dyads enrolled in the program for a year), there were improvements in some metrics for both patients and caregivers, despite the expected decline in cognitive function and ADL/IADL. As data is accrued, we anticipate it will identify patients and support resources that will be most beneficial.

